# Supplementary figures and images for: Coral growth along a natural gradient of seawater temperature, pH, and oxygen in a nearshore seagrass bed on Dongsha Atoll, Taiwan
Source: PLoS One. 2024 Oct 23;19(10):e0312263. doi: 10.1371/journal.pone.0312263 (PMC11498697; doi:10.1371/journal.pone.0312263)

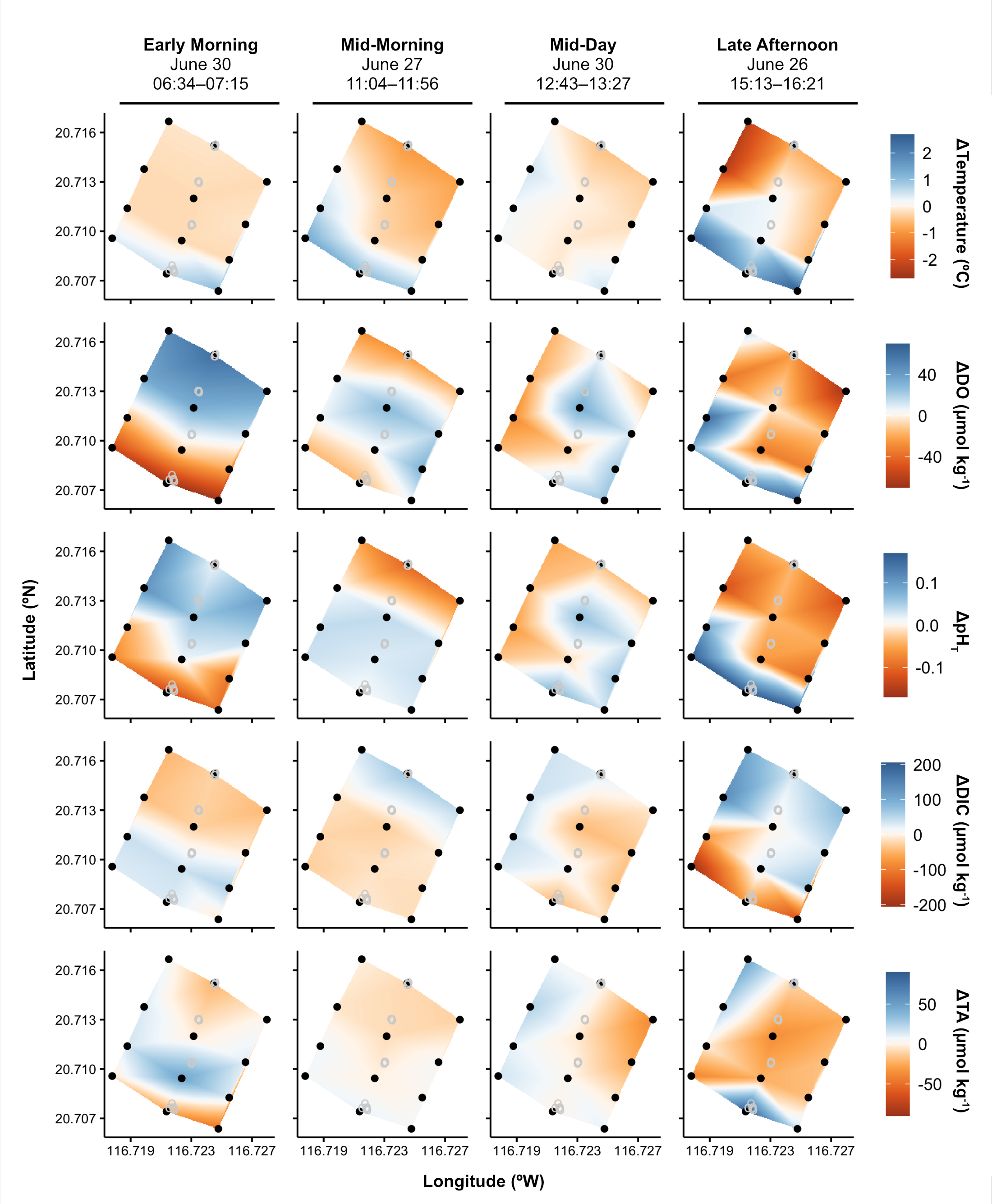

Supplement: S1 Fig — Spatial gradients in temperature (°C), dissolved oxygen (DO; μmol kg-1), total scale pH (pHT), dissolved inorganic carbon (DIC; μmol kg-1); and total alkalinity (TA; μmol kg-1) across the four spatial surveys (stations denoted by black circles) in the Dongsha Island north shore seagrass bed. Coral core collection locations are denoted by gray open circles. Times listed below dates represent the time of collection of the first and last sample of the survey (local time). Delta values reported were calculated as the difference between the survey mean and the value recorded at a given station (i.e., positive values indicate that the value recorded at that station was higher than the survey average and negative values indicate that the value recorded at the station was lower than the survey average). (TIFF) [file pone.0312263.s001.tiff]

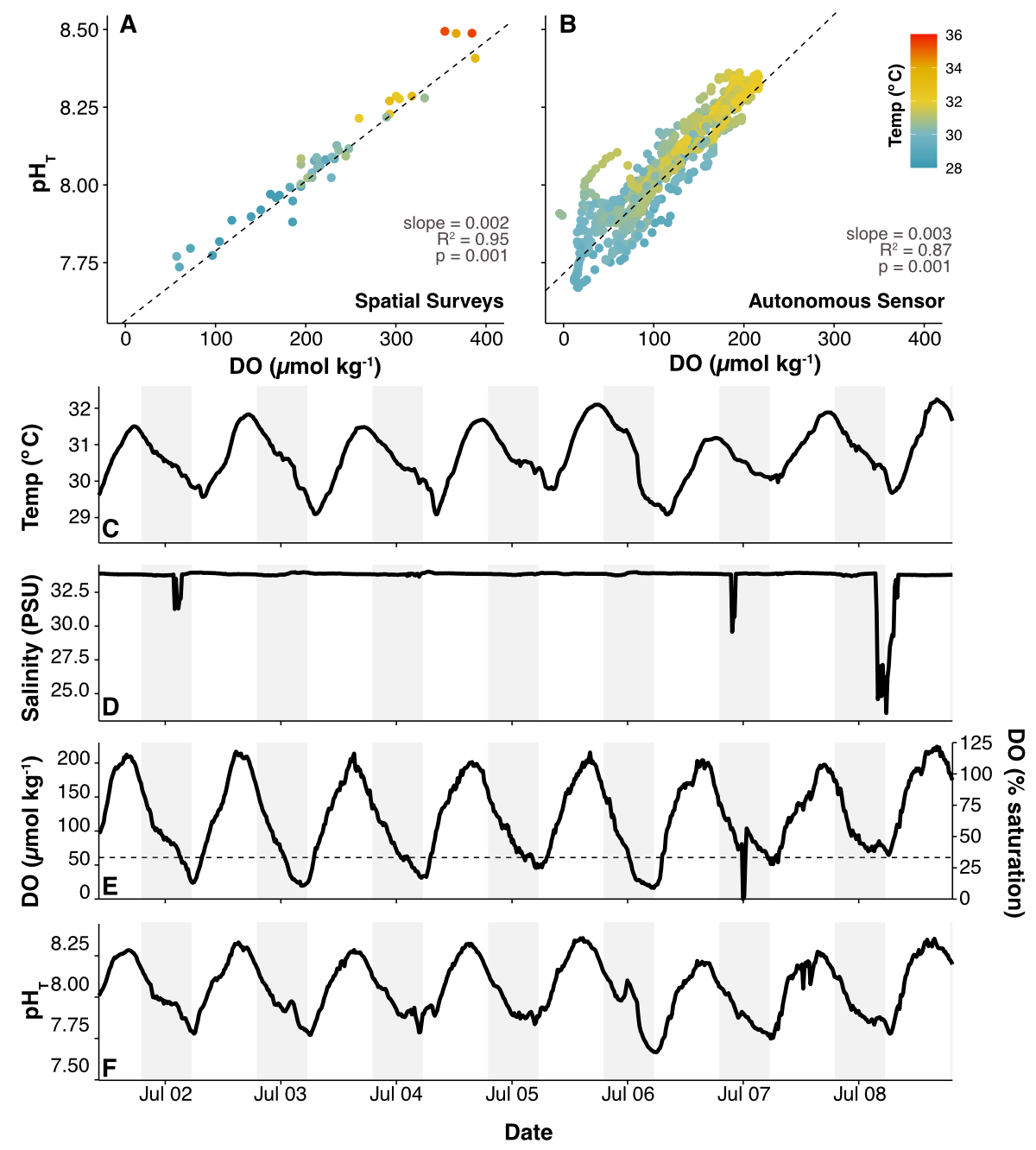

Supplement: S2 Fig — Relationship between total scale pH (pHT) and dissolved oxygen (DO; μmol kg-1) colored by temperature (°C) from the (A) spatial seawater surveys and (B) autonomous sensor deployed at a nearshore seagrass site including the slope, R2, and p-value from a linear regression. Time series of (C) temperature (°C), (D) salinity (PSU), (E) DO (μmol kg-1 [left] and percent saturation [right]), and (F) pHT from the IDRONAUT CTD sensor deployed in the shallow nearshore seagrass. Gray shaded boxes denote local nighttime hours. Dashed line in E denotes severe hypoxia threshold (61 μmol kg-1). (TIFF) [file pone.0312263.s002.tiff]

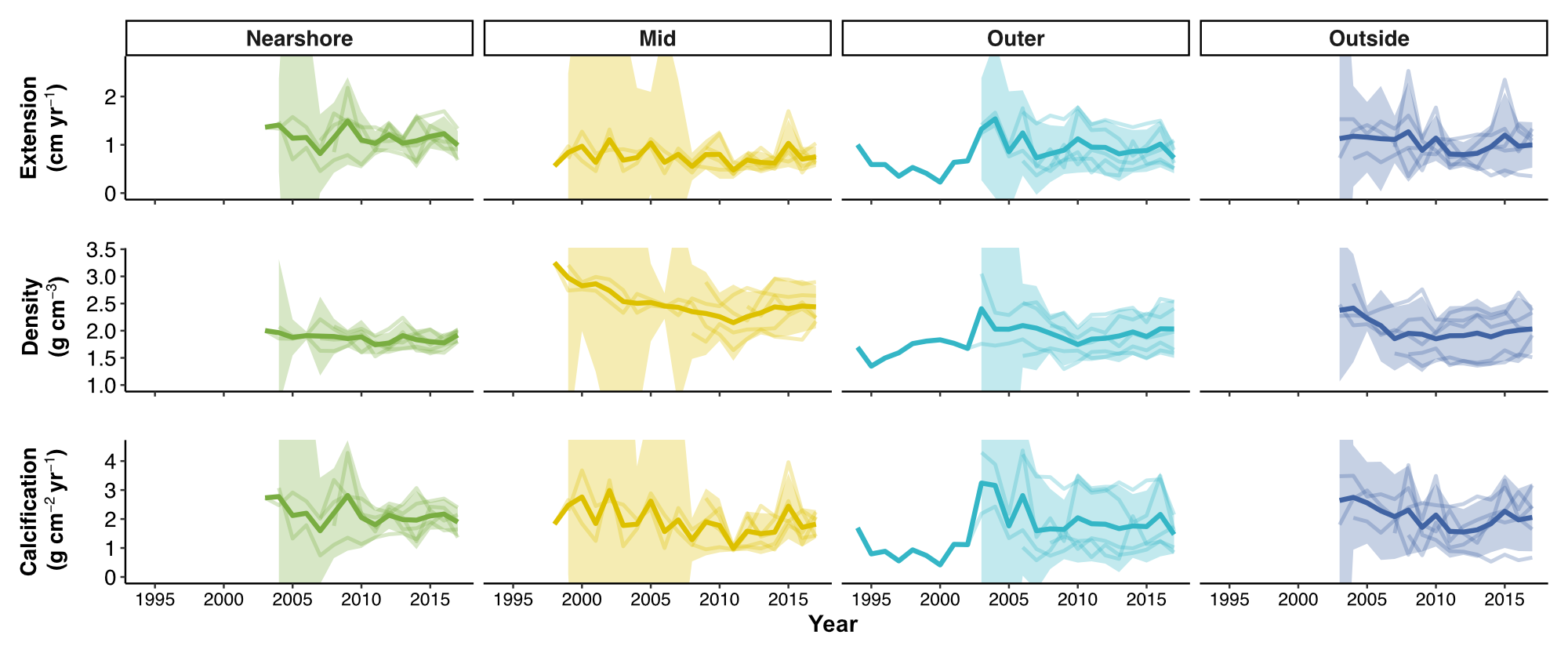

Supplement: S3 Fig — Time series of annual extension rate (cm year-1), density (g cm-3), and calcification rate (g cm-2 year-1) for all years and cores at each of the four collection locations (columns; n = 4 cores at nearshore, mid, and outer sites, and n = 3 at the outside site). Lighter color lines are time series for individual cores and the darker line is the mean for all cores at that site with 95% confidence interval shading above and below. (TIFF) [file pone.0312263.s003.tiff]
