# Supplementary material for: Coral growth along a natural gradient of seawater temperature, pH, and oxygen in a nearshore seagrass bed on Dongsha Atoll, Taiwan
Source: PLoS One. 2024 Oct 23;19(10):e0312263. doi: 10.1371/journal.pone.0312263 (PMC11498697; doi:10.1371/journal.pone.0312263)
Supplement: S1 Table — Minimum, maximum, range, and mean (± 1 standard deviation; n = 12 per survey) temperature, salinity, dissolved oxygen concentration, dissolved oxygen percent saturation, total scale pH (pHT), dissolved inorganic carbon, and total alkalinity for each spatial survey from in situ YSI measurements and bottle sample data. (DOCX) [file pone.0312263.s004.docx]

|  |  | | **Survey** | | | |
| --- | --- | --- | --- | --- | --- | --- |
|  |  | | **Early Morning**  June 30  06:34–07:15 | **Mid-Morning**  June 27  11:04–11:56 | **Mid-Day**  June 30  12:43–13:27 | **Late Afternoon**  June 26  15:13–16:21 |
| **Temperature**  **(ºC)** | | *Min* | 28.3 | 28.7 | 29.8 | 30.5 |
|  |  | *Max* | 29.6 | 31.0 | 30.8 | 35.5 |
|  |  | *Range* | 1.3 | 2.3 | 1.0 | 5.0 |
|  |  | *Mean ± SD* | 28.6 ± 0.4 | 29.7 ± 0.8 | 30.3 ± 0.3 | 32.9 ± 1.6 |
| **Salinity**  **(PSU)** | | *Min* | 34.3 | 32.6 | 34.3 | 33.5 |
|  |  | *Max* | 34.3 | 34.2 | 34.3 | 35.2 |
|  |  | *Range* | 0.0 | 1.6 | 0.0 | 1.7 |
|  |  | *Mean ± SD* | 34.3 ± 0.0 | 33.9 ± 0.5 | 34.3 ± 0.0 | 34.2 ± 0.4 |
| **Dissolved Oxygen**  **(µmol kg^-1^)** | | *Min* | 61.2 | 186.8 | 199.1 | 263.6 |
|  |  | *Max* | 189.7 | 244.9 | 260.3 | 392.8 |
|  |  | *Range* | 128.5 | 58.2 | 61.2 | 129.3 |
|  |  | *Mean ± SD* | 130.1 ± 45.3 | 214.4 ± 19.5 | 227.4 ± 20 | 330.6 ± 41.7 |
| **Dissolved Oxygen Percent Saturation (%)** | | *Min* | 32 | 95 | 103 | 142 |
|  |  | *Max* | 97 | 127 | 138 | 224 |
|  |  | *Range* | 65 | 32 | 35 | 82 |
|  |  | *Mean ± SD* | 67 ± 23 | 111 ± 10 | 120 ± 11 | 181 ± 26 |
| **pH_T_** | | *Min* | 7.73 | 7.94 | 7.99 | 8.2 |
|  |  | *Max* | 7.97 | 8.08 | 8.12 | 8.49 |
|  |  | *Range* | 0.24 | 0.14 | 0.13 | 0.29 |
|  |  | *Mean ± SD* | 7.86 ± 0.08 | 8.05 ± 0.05 | 8.05 ± 0.05 | 8.32 ± 0.11 |
| **Dissolved Inorganic Carbon**  **(µmol kg^-1^)** | | *Min* | 1952 | 1836 | 1819 | 1397 |
|  |  | *Max* | 2056 | 1933 | 1904 | 1739 |
|  |  | *Range* | 104 | 97 | 85 | 342 |
|  |  | *Mean ± SD* | 1999 ± 37 | 1865 ± 32 | 1863 ± 35 | 1601 ± 102 |
| **Total Alkalinity**  **(µmol kg^-1^)** | | *Min* | 2168 | 2178 | 2161 | 2107 |
|  |  | *Max* | 2263 | 2197 | 2223 | 2233 |
|  |  | *Range* | 95 | 20 | 63 | 126 |
|  |  | *Mean ± SD* | 2217 ± 24 | 2189 ± 7 | 2199 ± 17 | 2146 ± 37 |
