# Supplementary material for: Coral growth along a natural gradient of seawater temperature, pH, and oxygen in a nearshore seagrass bed on Dongsha Atoll, Taiwan
Source: PLoS One. 2024 Oct 23;19(10):e0312263. doi: 10.1371/journal.pone.0312263 (PMC11498697; doi:10.1371/journal.pone.0312263)
Supplement: S2 Table — Slope, intercept, R2, and p-value for regressions between salinity normalized total alkalinity (nTA) and dissolved inorganic carbon (nDIC) as well as dissolved oxygen (DO) and salinity normalized net community productivity derived from DIC (nNCPDIC) across all surveys and for individual surveys. Regression lines associated with these statistics for all surveys grouped (first column only) are plotted in Fig 4. Asterisks indicate level of significance (* p ≤ 0.05, ** p ≤ 0.01, *** p ≤ 0.001). (DOCX) [file pone.0312263.s005.docx]

|  |  | **All Surveys** | **Early Morning** | **Mid- Morning** | **Mid-**  **Day** | **Late Afternoon** |
| --- | --- | --- | --- | --- | --- | --- |
| **nTA ~ nDIC** | *slope* | 0.177 | 0.148 | 0.457 | 0.264 | 0.158 |
|  | *intercept* | 1864.364 | 1914.620 | 1346.829 | 1700.563 | 1893.706 |
|  | *R^2^* | 0.448 | 0.052 | 0.882 | 0.298 | 0.095 |
|  | *p-value* | 0.001*** | 0.256 | 0.003** | 0.038* | 0.150 |
| **DO ~ nDIC_NCP_** | *slope* | -0.513 | -1.023 | -0.258 | -0.563 | -0.327 |
|  | *intercept* | 1165.066 | 2157.027 | 696.688 | 1271.554 | 860.948 |
|  | *R^2^* | 0.883 | 0.679 | 0.367 | 0.756 | 0.654 |
|  | *p-value* | 0.001*** | 0.003** | 0.013* | 0.001*** | 0.001*** |
