# Supplementary material for: Coral growth along a natural gradient of seawater temperature, pH, and oxygen in a nearshore seagrass bed on Dongsha Atoll, Taiwan
Source: PLoS One. 2024 Oct 23;19(10):e0312263. doi: 10.1371/journal.pone.0312263 (PMC11498697; doi:10.1371/journal.pone.0312263)
Supplement: S3 Table — Daily statistics (mean daily minimum, maximum, range, and mean ± 1 SD) and deployment absolute minima and maxima for temperature, salinity, dissolved oxygen (DO) concentration, dissolved oxygen percent saturation, and total scale pH (pHT) recorded by the autonomous sensor in the shallow seagrass. Only full days (i.e., complete 24-hour cycle) are included in daily means (2–7 July 2018). (DOCX) [file pone.0312263.s006.docx]

|  | **Temp**  **(ºC)** | **Salinity**  **(PSU)** | **DO**  **(µmol kg^-1^)** | **DO Saturation**  **(%)** | **pH_T_** |
| --- | --- | --- | --- | --- | --- |
| **Mean daily min** | 29.4 ± 0.4 | 32.6 ± 1.8 | 20.7 ± 17.1 | 11 ± 9 | 7.76 ± 0.06 |
| **Mean daily max** | 31.7 ± 0.3 | 34.0 ± 0.0 | 208.1 ± 8.2 | 111 ± 4 | 8.30 ± 0.05 |
| **Mean daily range** | 2.3 ± 0.2 | 1.4 ± 1.8 | 187.5 ± 14.2 | 100 ± 7 | 0.53 ± 0.02 |
| **Mean daily mean** | 30.7 ± 0.3 | 33.8 ± 0.1 | 115.4 ± 5.0 | 61 ± 3 | 8.04 ± 0.04 |
| **Deployment min** | 29.1 | 23.6 | 0.0 | 0 | 7.67 |
| **Deployment max** | 32.2 | 34.0 | 224.1 | 121 | 8.36 |
