# Supplementary material for: Coral growth along a natural gradient of seawater temperature, pH, and oxygen in a nearshore seagrass bed on Dongsha Atoll, Taiwan
Source: PLoS One. 2024 Oct 23;19(10):e0312263. doi: 10.1371/journal.pone.0312263 (PMC11498697; doi:10.1371/journal.pone.0312263)
Supplement: S4 Table — Mean annual extension, density, and calcification rates between 2012 and 2017 for corals at the nearshore (n = 4), mid (n = 4), outer (n = 4), and outside sites (n = 3), as well as averaged across all sites (n = 15). Statistically significant differences between sites for extension, density, and calcification rates are indicated with superscript letters and asterisks indicating level of significance for each column (* p ≤ 0.05, ** p ≤ 0.01, *** p ≤ 0.001). (DOCX) [file pone.0312263.s007.docx]

|  | **Mean annual extension rate**  **(cm year^-1^)** | **Mean annual density**  **(g cm^-3^)** | **Mean annual calcification rate**  **(g cm^-2^ year^-1^)** |
| --- | --- | --- | --- |
| Nearshore | 1.10 ± 0.29^a^ | 1.79 ± 0.12 ^a^ | 1.95 ± 0.44^a^ |
| Mid | 0.69 ± 0.18^b*^ | 2.38 ± 0.39 ^b*^ | 1.66 ± 0.51^a^ |
| Outer | 0.94 ± 0.36^ab^ | 1.97 ± 0.38^ab^ | 1.93 ± 0.96^a^ |
| Outside | 1.01 ± 0.24^a*^ | 2.26 ± 0.17^b***^ | 2.28 ± 0.53^a^ |
| **Across all sites** | 0.93 ± 0.31 | 2.09 ± 0.38 | 1.93 ± 0.68 |
