# Supplementary material for: Coral growth along a natural gradient of seawater temperature, pH, and oxygen in a nearshore seagrass bed on Dongsha Atoll, Taiwan
Source: PLoS One. 2024 Oct 23;19(10):e0312263. doi: 10.1371/journal.pone.0312263 (PMC11498697; doi:10.1371/journal.pone.0312263)
Supplement: S5 Table — Slope, intercept, R2, and p-value for linear regressions of coral growth data from 2012 to 2017 for each site (Nearshore, Mid, Outer, Outside). Regression lines associated with these statistics are plotted in Fig 5D-5F (if significant, as denoted by asterisks: * p ≤ 0.05, ** p ≤ 0.01, *** p ≤ 0.001). (DOCX) [file pone.0312263.s008.docx]

|  |  | **Nearshore** | **Mid** | **Outer** | **Outside** |
| --- | --- | --- | --- | --- | --- |
| **extension ~ density** | *slope* | -1.450 | 0.044 | 0.532 | -0.310 |
|  | *intercept* | 3.698 | 0.591 | -0.103 | 1.713 |
|  | *R^2^* | 0.385 | 0.009 | 0.324 | 0.045 |
|  | *p-value* | 0.002** | 0.319 | 0.003* | 0.205 |
| **density ~ calcification** | *slope* | -0.118 | 0.457 | 0.305 | 0.025 |
|  | *intercept* | 2.019 | 1.623 | 1.378 | 2.205 |
|  | *R^2^* | 0.178 | 0.363 | 0.597 | 0.007 |
|  | *p-value* | 0.025* | 0.002** | 0.001*** | 0.344 |
| **calcification ~ extension** | *slope* | 1.484 | 2.456 | 2.593 | 2.106 |
|  | *intercept* | 0.316 | -0.045 | -0.518 | 0.151 |
|  | *R^2^* | 0.940 | 0.713 | 0.914 | 0.912 |
|  | *p-value* | 0.001*** | 0.001*** | 0.001*** | 0.001*** |
