# Supplementary material for: Coral growth along a natural gradient of seawater temperature, pH, and oxygen in a nearshore seagrass bed on Dongsha Atoll, Taiwan
Source: PLoS One. 2024 Oct 23;19(10):e0312263. doi: 10.1371/journal.pone.0312263 (PMC11498697; doi:10.1371/journal.pone.0312263)
Supplement: Attachment — Submitted filename: Response to Reviewers.docx [file pone.0312263.s010.docx]

**Response to Reviewers**

***Please note that all line numbers correspond to the line numbers in the manuscript file with track changes*

***Journal Requirements:***

1. When submitting your revision, we need you to address these additional requirements.

Please ensure that your manuscript meets PLOS ONE's style requirements, including those for file naming. The PLOS ONE style templates can be found at 
[https://journals.plos.org/plosone/s/file?id=wjVg/PLOSOne_formatting_sample_main_body.pdf](https://urldefense.com/v3/__https:/journals.plos.org/plosone/s/file?id=wjVg*PLOSOne_formatting_sample_main_body.pdf__;Lw!!Mih3wA!DL07q92qCcakHpcFtqBQeMXHDCpIV6-gzm4mrXuC2rTxAtBtifSzzBt9mJuQfahI3olR0W0r3B7ewVkb$) and 
[https://journals.plos.org/plosone/s/file?id=ba62/PLOSOne_formatting_sample_title_authors_affiliations.pdf](https://urldefense.com/v3/__https:/journals.plos.org/plosone/s/file?id=ba62*PLOSOne_formatting_sample_title_authors_affiliations.pdf__;Lw!!Mih3wA!DL07q92qCcakHpcFtqBQeMXHDCpIV6-gzm4mrXuC2rTxAtBtifSzzBt9mJuQfahI3olR0W0r3MqQcj8W$)

**Response**: We confirm that we have double-checked that the MS (and files) meet the linked style requirements.

2. Did you know that depositing data in a repository is associated with up to a 25% citation advantage ([https://doi.org/10.1371/journal.pone.0230416)?](https://urldefense.com/v3/__https:/doi.org/10.1371/journal.pone.0230416)?__;!!Mih3wA!DL07q92qCcakHpcFtqBQeMXHDCpIV6-gzm4mrXuC2rTxAtBtifSzzBt9mJuQfahI3olR0W0r3JZrn4Ix$) If you’ve not already done so, consider depositing your raw data in a repository to ensure your work is read, appreciated and cited by the largest possible audience. You’ll also earn an Accessible Data icon on your published paper if you deposit your data in any participating repository ([https://plos.org/open-science/open-data/#accessible-data](https://urldefense.com/v3/__https:/plos.org/open-science/open-data/*accessible-data__;Iw!!Mih3wA!DL07q92qCcakHpcFtqBQeMXHDCpIV6-gzm4mrXuC2rTxAtBtifSzzBt9mJuQfahI3olR0W0r3BwqOmTr$)).

**Response**: We have uploaded the data files to Zenodo (10.5281/zenodo.10892686) and the code to GitHub (https://github.com/apezner/DongshaSeagrassCores), which are both public repositories.

3. Please include a complete copy of PLOS’ questionnaire on inclusivity in global research in your revised manuscript. Our policy for research in this area aims to improve transparency in the reporting of research performed outside of researchers’ own country or community. The policy applies to researchers who have travelled to a different country to conduct research, research with Indigenous populations or their lands, and research on cultural artefacts. The questionnaire can also be requested at the journal’s discretion for any other submissions, even if these conditions are not met. 
Please find more information on the policy and a link to download a blank copy of the questionnaire here: [https://journals.plos.org/plosone/s/best-practices-in-research-reporting](https://urldefense.com/v3/__https:/journals.plos.org/plosone/s/best-practices-in-research-reporting__;!!Mih3wA!DL07q92qCcakHpcFtqBQeMXHDCpIV6-gzm4mrXuC2rTxAtBtifSzzBt9mJuQfahI3olR0W0r3NuJOO0b$). Please upload a completed version of your questionnaire as Supporting Information when you resubmit your manuscript.”

**Response**: We have completed the questionnaire and attached it as a Supporting Information file.

4. Thank you for stating in your Funding Statement: 
This research was supported by the National Science Foundation ([https://www.nsf.gov/](https://urldefense.com/v3/__https:/www.nsf.gov/__;!!Mih3wA!DL07q92qCcakHpcFtqBQeMXHDCpIV6-gzm4mrXuC2rTxAtBtifSzzBt9mJuQfahI3olR0W0r3ElSErDz$)) grants OCE-1255042 (AJA) and OCE-1829778 (AJA), a National Science Foundation Graduate Research Fellowship DGE-2038238 (AKP), and Philanthropic Educational Organization (P.E.O.; [https://www.peointernational.org/peo-scholar-awards](https://urldefense.com/v3/__https:/www.peointernational.org/peo-scholar-awards__;!!Mih3wA!DL07q92qCcakHpcFtqBQeMXHDCpIV6-gzm4mrXuC2rTxAtBtifSzzBt9mJuQfahI3olR0W0r3HKSebpG$)) Scholar Award (AKP). These funders did not play any role in study design, data collection and analysis, decision to publish, or preparation of the manuscript.

Please provide an amended statement that declares all the funding or sources of support (whether external or internal to your organization) received during this study, as detailed online in our guide for authors at [http://journals.plos.org/plosone/s/submit-now.](https://urldefense.com/v3/__http:/journals.plos.org/plosone/s/submit-now.__;!!Mih3wA!DL07q92qCcakHpcFtqBQeMXHDCpIV6-gzm4mrXuC2rTxAtBtifSzzBt9mJuQfahI3olR0W0r3JHMHgeX$)  Please also include the statement “There was no additional external funding received for this study.” in your updated Funding Statement. 
Please include your amended Funding Statement within your cover letter. We will change the online submission form on your behalf.

**Response**: The information provided in our original funding statement was correct as written. We added the requested text “There was no additional external funding received for this study” to the end of the statement. This is provided in the cover letter.

5. We noted in your submission details that a portion of your manuscript may have been presented or published elsewhere.

*The dissolved oxygen and temperature data only from the autonomous sensor in the seagrass was used in a recent publication by Pezner et al. in Nature Climate Change (2023), which compiled a global dataset of dissolved oxygen in reef habitats around the world. As this dataset is presented in full in the current MS (temperature, pH, dissolved oxygen, salinity) and is only a small part of the current analysis, we do not believe this constitutes dual publication. These data are only included in the present manuscript only to illustrate the temporal variability in environmental and chemical parameters that the corals are exposed to, but do not constitute the main findings of this study*.

Please clarify whether this [conference proceeding or publication] was peer-reviewed and formally published. If this work was previously peer-reviewed and published, in the cover letter please provide the reason that this work does not constitute dual publication and should be included in the current manuscript.

**Response**: We have addressed this in the cover letter with further explanation.

6. When completing the data availability statement of the submission form, you indicated that you will make your data available on acceptance. We strongly recommend all authors decide on a data sharing plan before acceptance, as the process can be lengthy and hold up publication timelines. Please note that, though access restrictions are acceptable now, your entire data will need to be made freely accessible if your manuscript is accepted for publication. This policy applies to all data except where public deposition would breach compliance with the protocol approved by your research ethics board. If you are unable to adhere to our open data policy, please kindly revise your statement to explain your reasoning and we will seek the editor's input on an exemption. Please be assured that, once you have provided your new statement, the assessment of your exemption will not hold up the peer review process.

**Response**: We have uploaded the data files to Zenodo (10.5281/zenodo.10892686) and the code to GitHub (https://github.com/apezner/DongshaSeagrassCores), which are both public repositories. This statement appears in the Manuscript on Lines 436-438.

7. We note that Figure 1 in your submission contain map and satellite images which may be copyrighted. All PLOS content is published under the Creative Commons Attribution License (CC BY 4.0), which means that the manuscript, images, and Supporting Information files will be freely available online, and any third party is permitted to access, download, copy, distribute, and use these materials in any way, even commercially, with proper attribution. For these reasons, we cannot publish previously copyrighted maps or satellite images created using proprietary data, such as Google software (Google Maps, Street View, and Earth). For more information, see our copyright guidelines: [http://journals.plos.org/plosone/s/licenses-and-copyright](https://urldefense.com/v3/__http:/journals.plos.org/plosone/s/licenses-and-copyright__;!!Mih3wA!DL07q92qCcakHpcFtqBQeMXHDCpIV6-gzm4mrXuC2rTxAtBtifSzzBt9mJuQfahI3olR0W0r3Pbqzl7A$).
 
We require you to either present written permission from the copyright holder to publish these figures specifically under the CC BY 4.0 license, or remove the figures from your submission:
 
a. You may seek permission from the original copyright holder of Figure 1 to publish the content specifically under the CC BY 4.0 license.  
 
We recommend that you contact the original copyright holder with the Content Permission Form ([http://journals.plos.org/plosone/s/file?id=7c09/content-permission-form.pdf](https://urldefense.com/v3/__http:/journals.plos.org/plosone/s/file?id=7c09*content-permission-form.pdf__;Lw!!Mih3wA!DL07q92qCcakHpcFtqBQeMXHDCpIV6-gzm4mrXuC2rTxAtBtifSzzBt9mJuQfahI3olR0W0r3F0PiwKE$)) and the following text:
“I request permission for the open-access journal PLOS ONE to publish XXX under the Creative Commons Attribution License (CCAL) CC BY 4.0 ([http://creativecommons.org/licenses/by/4.0/](https://urldefense.com/v3/__http:/creativecommons.org/licenses/by/4.0/__;!!Mih3wA!DL07q92qCcakHpcFtqBQeMXHDCpIV6-gzm4mrXuC2rTxAtBtifSzzBt9mJuQfahI3olR0W0r3LrEUmVG$)). Please be aware that this license allows unrestricted use and distribution, even commercially, by third parties. Please reply and provide explicit written permission to publish XXX under a CC BY license and complete the attached form.”
 
Please upload the completed Content Permission Form or other proof of granted permissions as an "Other" file with your submission.
 
In the figure caption of the copyrighted figure, please include the following text: “Reprinted from [ref] under a CC BY license, with permission from [name of publisher], original copyright [original copyright year].”
 
b. If you are unable to obtain permission from the original copyright holder to publish these figures under the CC BY 4.0 license or if the copyright holder’s requirements are incompatible with the CC BY 4.0 license, please either i) remove the figure or ii) supply a replacement figure that complies with the CC BY 4.0 license. Please check copyright information on all replacement figures and update the figure caption with source information. If applicable, please specify in the figure caption text when a figure is similar but not identical to the original image and is therefore for illustrative purposes only.
The following resources for replacing copyrighted map figures may be helpful:
 
USGS National Map Viewer (public domain): [http://viewer.nationalmap.gov/viewer/](https://urldefense.com/v3/__http:/viewer.nationalmap.gov/viewer/__;!!Mih3wA!DL07q92qCcakHpcFtqBQeMXHDCpIV6-gzm4mrXuC2rTxAtBtifSzzBt9mJuQfahI3olR0W0r3DN9PIJD$)
The Gateway to Astronaut Photography of Earth (public domain): [http://eol.jsc.nasa.gov/sseop/clickmap/](https://urldefense.com/v3/__http:/eol.jsc.nasa.gov/sseop/clickmap/__;!!Mih3wA!DL07q92qCcakHpcFtqBQeMXHDCpIV6-gzm4mrXuC2rTxAtBtifSzzBt9mJuQfahI3olR0W0r3EG6jCF5$)
Maps at the CIA (public domain): [https://www.cia.gov/library/publications/the-world-factbook/index.html](https://urldefense.com/v3/__https:/www.cia.gov/library/publications/the-world-factbook/index.html__;!!Mih3wA!DL07q92qCcakHpcFtqBQeMXHDCpIV6-gzm4mrXuC2rTxAtBtifSzzBt9mJuQfahI3olR0W0r3FkNIWhK$) and [https://www.cia.gov/library/publications/cia-maps-publications/index.html](https://urldefense.com/v3/__https:/www.cia.gov/library/publications/cia-maps-publications/index.html__;!!Mih3wA!DL07q92qCcakHpcFtqBQeMXHDCpIV6-gzm4mrXuC2rTxAtBtifSzzBt9mJuQfahI3olR0W0r3FDtNTdT$)
NASA Earth Observatory (public domain): [http://earthobservatory.nasa.gov/](https://urldefense.com/v3/__http:/earthobservatory.nasa.gov/__;!!Mih3wA!DL07q92qCcakHpcFtqBQeMXHDCpIV6-gzm4mrXuC2rTxAtBtifSzzBt9mJuQfahI3olR0W0r3DIYg4U9$)
Landsat: [http://landsat.visibleearth.nasa.gov/](https://urldefense.com/v3/__http:/landsat.visibleearth.nasa.gov/__;!!Mih3wA!DL07q92qCcakHpcFtqBQeMXHDCpIV6-gzm4mrXuC2rTxAtBtifSzzBt9mJuQfahI3olR0W0r3LZ4-BHT$)
USGS EROS (Earth Resources Observatory and Science (EROS) Center) (public domain): [http://eros.usgs.gov/#](https://urldefense.com/v3/__http:/eros.usgs.gov/*__;Iw!!Mih3wA!DL07q92qCcakHpcFtqBQeMXHDCpIV6-gzm4mrXuC2rTxAtBtifSzzBt9mJuQfahI3olR0W0r3PUOPq0V$)
Natural Earth (public domain): [http://www.naturalearthdata.com/](https://urldefense.com/v3/__http:/www.naturalearthdata.com/__;!!Mih3wA!DL07q92qCcakHpcFtqBQeMXHDCpIV6-gzm4mrXuC2rTxAtBtifSzzBt9mJuQfahI3olR0W0r3DOJgAYS$)

**Response**: While we appreciate the suggestions, none of the linked sources have satellite imagery of our specific site or of high enough quality to match the Google Earth images we previously used. We decided to use another source for these images (Planet Labs PBC). Planet Labs’ legal team has signed the required release form which has been attached to this resubmission.

***Additional Editor Comments:***

**EC1:** Dear Author,

both the reviewer and I have evaluated your manuscript. The manuscript is comprehensible. However, as indicated in reviewer comments , I advise considering the publication of a significantly more concise version. Several figures and tables could be moved to the supplement, and the text, particularly in the discussion section, can be substantially reduced (the reviewer has provided some suggestions, but an overall rigorous edit is necessary). The primary value lies in the data, which merit publication. Nevertheless, to align with the standards of PLOS One, the paper should be concise, with the majority of information presented in the supplement.
after this deep revision the manuscript will deserve publication

best egrads
Silvia Mazzuca
Handling Editor

**EC1 Response:** Thank you for your suggestions. We have re-organized the manuscript according to the Editor and Reviewer’s suggestions and moved Figures 5 and 6 as well as all Tables to the Supporting Information file. We have significantly reduced the text throughout the manuscript and especially in the Discussion. Please see below for specific editorial changes as they apply to specific comments. We believe these rigorous edits have improved the manuscript, while retaining key findings and data.

***Reviewer’s Comments to the Author***

**RC1.** Is the manuscript technically sound, and do the data support the conclusions?

The manuscript must describe a technically sound piece of scientific research with data that supports the conclusions. Experiments must have been conducted rigorously, with appropriate controls, replication, and sample sizes. The conclusions must be drawn appropriately based on the data presented.

Reviewer #1: Yes

**RC1 Response:** Thank you for your positive assessment.

**RC2**. Has the statistical analysis been performed appropriately and rigorously?

Reviewer #1: Yes

**RC2 Response:** Thank you for your positive assessment.

**RC3**. Have the authors made all data underlying the findings in their manuscript fully available?

The [PLOS Data policy](https://urldefense.com/v3/__http:/www.plosone.org/static/policies.action*sharing__;Iw!!Mih3wA!DL07q92qCcakHpcFtqBQeMXHDCpIV6-gzm4mrXuC2rTxAtBtifSzzBt9mJuQfahI3olR0W0r3JcLY3S0$) requires authors to make all data underlying the findings described in their manuscript fully available without restriction, with rare exception (please refer to the Data Availability Statement in the manuscript PDF file). The data should be provided as part of the manuscript or its supporting information, or deposited to a public repository. For example, in addition to summary statistics, the data points behind means, medians and variance measures should be available. If there are restrictions on publicly sharing data—e.g. participant privacy or use of data from a third party—those must be specified.

Reviewer #1: No

**RC3 Response:** We have provided the links to the data files on Zenodo (10.5281/zenodo.10892686) and the code files on GitHub (https://github.com/apezner/DongshaSeagrassCores) on Lines 436-438. These are both publicly available now.

**RC4**. Is the manuscript presented in an intelligible fashion and written in standard English?

PLOS ONE does not copyedit accepted manuscripts, so the language in submitted articles must be clear, correct, and unambiguous. Any typographical or grammatical errors should be corrected at revision, so please note any specific errors here.

Reviewer #1: Yes

**RC4 Response:** Thank you for your positive assessment.

**RC5**. Review Comments to the Author

Please use the space provided to explain your answers to the questions above. You may also include additional comments for the author, including concerns about dual publication, research ethics, or publication ethics. (Please upload your review as an attachment if it exceeds 20,000 characters)

Reviewer #1: Review- Manuscript Number: PONE-D-23-37336
‘Coral growth along a natural gradient of seawater temperature, pH, and oxygen in a nearshore seagrass bed on Dongsha Atoll, Taiwan’

The manuscript describes a well-executed and technically sound study that describes spatial patterns in water chemistry (DO, pH, DIC, TA, Temp) across an inshore-offshore gradient around Dongsha Island. The study site is an interesting habitat with a mixed seagrass and coral community and well-suited to test the hypothesis that primary producers convey an “ameliorating effect” to coral performance under increased CO2 availability. However, the study was only over a very short period of time, a week in June 2018. This limits its value, especially with regard to being able to interpret the data from the Porites cores as the main indicators of coral performance at this site.

The manuscript clearly complements earlier work, which conducted more measurements of water chemistry in the seagrass beds at the study site, Dongsha Island [eg ref 55, study over a year in 2011] and analysed coral cores in a neighbouring non-seagrass habitat [eg ref 58, collected in 2015, analysed 2007-2012]. To increase the value of the MS, I suggest that the authors more clearly outline how the current study builds on and/or complements this earlier work, and perhaps even consider presenting some of the earlier data in tables or analyses.

**RC5 Response:** Thank you for your suggestions. We note that while previous work has involved study of seagrass biology (refs 48, 49) and seagrass carbonate chemistry at one point in the seagrass (refs 43, 63), this study is the first to look at the variability in the biogeochemistry of the seagrass bed across both time and spatial scales. These combined scales allowed us to reveal significant inshore to offshore gradients that have not been previously shown at this site, and indeed at few other seagrass sites around the world (but see, 24). Moreover, this study is the first to pair coral core data with these in situ environmental measurements, to address how these highly variable conditions might affect coral growth. We have added text to the Discussion and Conclusion (Lines 789-791; 1283-1286) to further clarify how this study is unique and valuable even when compared to previous studies.

“Here, we reveal significant gradients in seawater temperature, dissolved oxygen (DO), and carbonate chemistry across the Dongsha seagrass bed from inshore to offshore during our study in the summer of 2018 that have not been previously captured.”

“While previous work at this site has explored carbonate chemistry variability in the seagrass bed, the present study is the first to examine the inshore to offshore gradient at fine spatial scales and pair this environmental data with biological data from corals living within these strong gradients.”

**RC6:** As it stands, the MS is descriptive and its value is in the data (which I understand will be publicly available at publication), which others may use for future studies or metanalyses. For this, the MS is publishable (after revision) - but my recommendation is to publish a much cut-back version (see some suggestions below in my detailed comments).

In my opinion, it is likely that the relatively young Porites colonies studied here are tolerant (eg selection during recruitment) to the local, diurnally highly variable, seawater chemistry (which is driven by the seagrass metabolism at 3 of the 4 sites). Hence, it is not surprising that the differences in Porites calcification, extension and density were less pronounced than expected by the authors. As the authors correctly conclude, other variables than the measured might have influenced the observed patterns. Also, interactions between the various environmental variables are not well understood and could also have an influence.

The discussion occasionally strays into speculation, and I recommend the authors take care not to over-interpret the limited available environmental data.

**RC6 Response:** Thank you for recognizing the value of this dataset. We have taken your comments here and below into consideration and have significantly reduced the Discussion word count to make it more concise and avoid speculation. Specifically, we trimmed the body of the paper for clarity and also cut many of the speculative paragraphs that were originally toward the end of the Discussion section on the coral cores. We have also added text that relates to your suggestions that the *Porites* in this seagrass bed are likely tolerant of these conditions, perhaps explaining why growth was not reduced at this site on Lines 1274-1280.

*“Regardless, the maintenance of consistent calcification rates across the chemical gradients in the seagrass bed suggest that the corals in the Dongsha seagrass have naturally acclimated to living in these conditions (or were selected for over time) and have thus been able to maintain their calcification rates. Being one of the only coral species to inhabit the seagrass bed suggests that these massive Porites may be particularly resistant to environmental stressors compared to other species found on Dongsha Atoll.”*

**RC7:** As an aside: The coral core data analysis was limited to only the years 2012-2017 (for QA reasons)- this for example does not include periods of potential early stages of recovery from the 2007 bleaching event which was record in (most?) analysed cores. This would have been interesting as the water chemistry at the 4 locations might have modulated the recovery trajectory.

**RC7 Response:** We agree that it would be interesting to include more data from the 2007 bleaching event from these cores. As you mention, we had excluded years prior to 2012 from the data analysis, as some cores (3/15) only had usable bands beginning in 2012 and we wanted all analyses to compare the same years. However, 12 of the 15 cores do have bands going back to at least 2007 or earlier. We note in the Discussion on Lines 1137-1143 that 4 of these cores exhibited high density stress bands in the year 2007, suggesting that they did experience stress and recover after 2007. However, we performed additional statistical tests looking for differences between bleaching years and non-bleaching years in this subset of cores and found no significant differences, thus we did not any further exploration of this subset of the data in the final version of the MS.

*Detailed comments:*

Introduction:
**RC8:** Note that correct citation for ref [5] is: Souter, D., Planes, S., Wicquart, J., Logan, M., Obura, D., Staub, F. (eds) (2021). Status of coral reefs of the world: 2020 report. Global Coral Reef Monitoring Network (GCRMN) and International Coral Reef Initiative (ICRI). DOI: 10.59387/WOTJ9184.

**RC8 Response:** Our apologies for the error, thank you for the correct citation. This has been amended on Lines 1530-1532.

**RC9:** Ref [6] is quite dated, lots of more recent refs after the 2015-17 bleaching

**RC9 Response:** We have replaced this reference with a more recent one (Lines 1533-1534):

Sully S, Burkepile DE, Donovan MK, Hodgson G, van Woesik R. A global analysis of coral bleaching over the past two decades. Nat Commun. 2019 Mar 20;10(1):1264.

Methods:
**RC10:** Describe somewhere how abundant the corals (esp Porites) are at your sites.

**RC10 Response:** Unfortunately, there is no quantitative data on coral abundance at our sites nor did we take strategic photos for benthic cover quantification, so we do not feel comfortable providing any numerical assessment especially by site. The presence of the corals in the seagrass in general can be viewed in Figure 1C. We attempted to estimate using satellite images but given the wide range in colony size, we did not feel that any quantitative assessment using this method would be accurate.

**RC11:** Line 206: write out NCP when first used here

**RC11 Response:** Thank you for catching this, we have expanded NCP to Net Community Production on Line 336.

**RC12:** Depths reported should be normalised to an appropriate tide datum (eg line 240/241), eg LAT

**RC12 Response:** We have corrected the depths to reflect depth relative to the mean lower low water based on available tide data from Dongsha Atoll. Given the small tidal range at Dongsha during the study period and the accuracy of our dive computer depth, the previously estimated depths are still accurate (see Lines 376-381).

**RC13:** Line :253 reword, sloppy language” Cores were slabbed”

**RC13 Response:** We have re-worded the phrasing of this sentence on Lines 400-403.

*“Cores were cut using a double-bladed lapidary saw to produce an 8 mm thick cross section from the center of each core that was then x-rayed with matching aluminum and aragonite (Tridacna sp. shell) wedges at 35 kVp and 0.3 mAs for 40 seconds using a Faxitron X-Ray in the SIO Marine Vertebrate Collection.”*

Results:
**RC14:** Line 301: suggest that Table 1 is moved to supplement

**RC14 Response:** Thank you for this suggestion. All tables have been moved to the Supporting Information, including what was previously Table 1 (now S1 Table). All references to this table have been changed accordingly.

**RC15:** Line 344/345: not so surprising, this is just a point in time and v localised factors could be at play here…

**RC15 Response:** Yes, we agree that with so few data points it is reasonable that the observations would reveal some unexpected variability.

**RC16:** Line 403: hypoxia every end of the night/before sunrise, not “evening”

**RC16 Response:** Yes, you are correct, thank you for the suggestion the change has been made on Lines 643-644.

**RC17:** Line 405: Fig 5- I would recommend gray shading for nighttime hours to make the graph more intuitive…

**RC17 Response:** Thank you for the advice, we have adjusted the Figure as suggested (now S2 Figure).

**RC18:** I find in Fig 5 the correlation between DO and pH and colour coded by temperature a bit misleading as it is perhaps more time of day and photosynthesis influencing this, as clearly illustrated in panel 5 c to f…

**RC18 Response:** (Note that this is now S2 Figure) We agree that the relationship between DO and pH is also influenced by the time of day (i.e., light availability) and balance of photosynthesis vs. respiration. Time of day, temperature, and photosynthetic/respiration rates are all highly correlated in this system (and most shallow marine systems – for example see the below references), thus we colored these points by temperature as a proxy for both time of day as well as net photosynthesis. Similarly, cooler temperatures, nighttime, and respiration rates are all linked in this system. As also illustrated in Panel C, temperature varies strongly with time of day (and daylight hours), thus we do not believe this graphical choice is ‘misleading’ as these factors are all tightly linked.

Cryer, S.E., Evans, C., Fowell, S.E., Andrews, G., Brown, P., Carvalho, F., Degallerie, D., Ludgate, J., Rosado, S., Sanders, R. and Strong, J.A., 2023. Characterizing Reef Net Metabolism Via the Diel Co‐Variation of pH and Dissolved Oxygen From High Resolution in Situ Sensors. *Global Biogeochemical Cycles*, *37*(9), p.e2022GB007577.

Cyronak, T., Takeshita, Y., Courtney, T.A., DeCarlo, E.H., Eyre, B.D., Kline, D.I., Martz, T., Page, H., Price, N.N., Smith, J. and Stoltenberg, L., 2020. Diel temperature and pH variability scale with depth across diverse coral reef habitats. *Limnology and Oceanography Letters*, *5*(2), pp.193-203.

**RC19:** Line 413: suggest that Table 1 is moved to supplement

**RC19 Response:** In line with RC14, Table 1 (and all other Tables) have been moved to the Supplement.

**RC20:** Line 421 and para below: Fig 6- what is reason for v high variability in parts of record?

**RC20 Response:** (Note this is now S3 Figure) - The wide confidence intervals in some years are a result of there being few corals with growth bands in those years (with dissimilar extension, density, or calcification rates – leading to large CIs). In terms of variation year to year in individual corals, we cannot say for sure what caused high variability in these growth metrics. We speculate that environmental conditions may have been more favorable in one year compared to another leading to lows and highs in these metrics, but without long-term in-situ environmental data it is difficult to determine the cause with certainty.

**RC21:** Line 449: omit referring to Table 3 when describing the statistical comparison between cores, this information is not in Table 3 (but should be). For this section I suggest that Fig 7 (d - f) Table 3 contains sufficient information (if statistical results are added as eg asterisks) to support the results and Fig 6 and Fig 7 a-c could be included in the supplementary material w/o losing much content.

**RC21 Response:** Thank you for pointing out this discrepancy and for your suggestions. We have added statistical information to what is now S4 Table as suggested. We have decided to move what was previously Figures 5 and 6 to the Supporting Information file (now S2 and S3 Figs). What was Figure 7 is now Figure 5 in the main MS. Given that the detailed statistical information is now in the SI, we opted to keep this figure in the main text to highlight the differences in growth parameters between locations (Fig 5 A,B,C) as well as the linear regressions between the different pairs of growth parameters (Fig 5 D,E,F), as these are our main findings from the cores.

Discussion:
**RC22:** Line 497: the SSP predictions are global averages- comparing the locally measured dial ranges of values to the predictions is not meaningful.

**RC22 Response:** We understand your concern and therefore have removed this comparison.

**RC23:** Line 504: suggestion for rewording “(applying published thresholds of < 61 µmol O2 kg-1or 2 mg L-1504 ; [35])”

**RC23 Response:** Thank you for the suggestion, we have added this on Lines 817-820:

“Notably, DO concentrations as measured by the CTD were severely hypoxic (applying a published threshold of < 61 µmol O2 kg-1 or 2 mg L-1; [35]) every single night of the deployment (S2 Fig).”

**RC24:** Line 507: “Compared to traditional reef environments, these regular hypoxic conditions are unique, but are nonetheless projected to increase in duration, frequency, and intensity as the oceans continue to warm and deoxygenate [4].” - what do you consider “traditional reef environments”, suggest something like: ‘’coral reef communities without substantial seagrass biomass’. Ref [4] is predictions for coral reef environments.

**RC24 Response:** We understand the potential confusion of this wording and thus have clarified this to “reef environments dominated mainly by stony corals” on Lines 822-823. We note that Ref 4 includes data on a variety of coral habitats in addition to “traditional” reefs (including some of the instrument data presented in this MS and one other seagrass bed hosting corals in the South China Sea).

**RC25:** Line 519 onwards: Some of the referenced studies appear to be from just seagrass beds not a mixed community such as the one studied here. Are there indications that the primary producers dominate the water chemistry in mixed communities and the fact that some corals are present is not expected to make much of a measurable difference (hence comparisons to seagrass-only habitats is valid)?

**RC25 Response:** There is unfortunately a quite limited literature focusing on the biogeochemistry of mixed seagrass communities that include large coral colonies similar to our site, which is one reason why we believe our data and findings fill a necessary gap in the field. We agree that primary producer-dominated environments such as seagrass beds or mangrove lagoons are already such highly productive ecosystems compared to traditional reef systems (e.g., Camp et al., 2016 [ref 39]) that the presence of corals in this seagrass bed would not elevate the productivity noticeably (and thus this comparison is valid).

**RC26:**Para 526-543: discussion here overly convoluted, the points here could be made more succinctly.

**RC26 Response:** We have significantly edited the Discussion to make it more concise, including removing much of this section.

**RC27:** Line 527: suggest “more pronounced” instead of “heightened”

**RC27 Response:** Thank you for the suggestion, we have made this change (Line 897).

**RC28:** Lines 528- 530: normalise to tide reference

**RC28 Response:** This sentence has been removed but we have done this in other sections where relevant (e.g., Lines 379-381).

**RC29:** Lines 530-532: “almost complethey covered”- as you have no data on the seagrass biomass I suggest you are bit more descriptive here- close to 100% cover of dense seagrass vs some sort of description of what the ‘outside’ seagrass community looked like, in comparison.

**RC29 Response:** We have changed this to on Lines 899-902:

“The nearshore stations were almost completely covered in seagrass (> 80 % cover of seagrass [55]), which led to a higher biomass to water volume ratio compared to the deeper outside stations (which contained mainly sand with some small patches of seagrass).”

**RC30:** Line 535: depth as a property is in most cases a proxy for variety of env variables that change along deoth gradients: eg benthic light, temperature, volume of overlying water, importance of gas exhancge at surface etc.

**RC30 Response:** Yes, we agree. We have added “and thus the many environmental properties that scale with depth such as light, gas exchange, temperature, etc.” to this sentence to clarify this point on Lines 953-954.

**RC31:** Line 537: Not sure what you mean by “path of the water over the reef”- you mentioned the relevant variables, flow speed, residence time (which are also related)

**RC31 Response:** The path of the water refers to the history and direction of the movement of water over the reef, which is also very important as the direction of flow (in addition to the speed and residence time) influences the chemical properties of the seawater as it moves over an area from the start of its path to the end of its path. We have clarified this in the sentence as “the trajectory of water flow over the reef” on Line 956.

**RC32:** Line 538: “modify the above seawater”- unusual expression, this is just physics, ie area of seafloor vs overlying water volume, and diffusion/dispersion of metabolites…

**RC32 Response:** We agree that this is the result of interactions between physics and biology on the reef. We note, however, that this is common terminology used in the coral reef physical oceanographic and biogeochemical fields, for example see:

Anthony, K.R., A. Kleypas, J. and Gattuso, J.P., 2011. Coral reefs modify their seawater carbon chemistry–implications for impacts of ocean acidification. Global Change Biology, 17(12), pp.3655-3666.

Kleypas, J.A., Anthony, K.R. and Gattuso, J.P., 2011. Coral reefs modify their seawater carbon chemistry–case study from a barrier reef (Moorea, French Polynesia). Global change biology, 17(12), pp.3667-3678.

Unsworth, R.K., Collier, C.J., Henderson, G.M. and McKenzie, L.J., 2012. Tropical seagrass meadows modify seawater carbon chemistry: implications for coral reefs impacted by ocean acidification. Environmental Research Letters, 7(2), p.024026.

**RC33:** Line 574 (and elsewhere): “seagrass corals”- sloppy term…

**RC33 Response:** We have edited this phrasing to “seagrass bed *Porites*” where appropriate in the MS on Lines 1044, 1049, 1052-53, 1054, 1057, 1128, 1138-39, 1244, 1254, and 1489.

**RC34:** Line 598-600: this is speculation

**RC34 Response:** This sentence has been removed from the revised MS.

**RC35:** Line 611: suggest to add “meadow” or “bed” to ‘seagrass’ and saying Porites instead of ‘corals’

**RC35 Response:** Thank you for the suggestion, we have changed this where needed as noted in the RC33 response.

**RC36:** Line 613/614: suggest: “Porites at the outer sites” and “cores from the mid and outer sites”

**RC36 Response:** Thank you for the suggestion, we have changed this as proposed on Lines 1146-1236:

*“…particularly for the Porites at the outer site (Fig 5A-C), we did observe some significant differences in annual extension rate and annual density between the cores from the nearshore site and the cores from the mid and outside sites (Fig 5A-C). Notably, we found that the cores from both the nearshore and outside sites had significantly higher mean annual extension rates than the cores from the mid site…”*

**RC37:** Line 660 & Line 680: Porites appears to be particularly tolerant to a many env variables, not just low pH/high DIC

**RC37 Response:** This sentence was removed in the revised MS.

**RC38:** Line 682: Consider adding this reference, showing that a previously reported decline in calcification in GBR Porites was only transitory:
Cantin NE, Lough JM (2014) Surviving Coral Bleaching Events: Porites Growth Anomalies on the Great Barrier Reef. PLoS ONE 9: e88720 doi 10.1371/journal.pone.0088720

**RC38 Response:** Thank you for bringing this study to our attention, but the relevant sentence was removed in this version of the MS.

**RC39:** Line 692: delete “and dissolved oxygen concentrations”, next sentence explains the changes in water chemistry you measured.

**RC39 Response:** We have made this change on Line 1287.
